# Hilar Bile Duct Granuloma Misdiagnosed As Hilar Cholangiocarcinoma: A Case Report

**DOI:** 10.7759/cureus.85583

**Published:** 2025-06-09

**Authors:** Feng Wei, Zhou Fan, Xiao Mengzhi, Yu Jun, Tan Xingguo

**Affiliations:** 1 Hepatobiliary Surgery, Yueyang Hospital (Affiliated to Hunan Normal University), Yueyang, CHN

**Keywords:** granuloma, hilar bile duct, misdiagnosis, schistosoma, surgery

## Abstract

Schistosomiasis is the second most common human parasitic infection after malaria. The granulomatous inflammatory damage to the liver caused by its eggs and the resulting liver fibrosis are the basic characteristics of schistosomiasis and also the main cause of death. We report an elderly male patient from an endemic area who presented with abdominal distension and jaundice. He was initially diagnosed with hilar cholangiocarcinoma, but postoperative biopsy indicated schistosomal granulomatous inflammation. This case has provided us with a deeper understanding of the diagnosis and treatment of hilar schistosomal granuloma.

## Introduction

Schistosomiasis is the second most common human parasitic infection after malaria [1]. The core pathological feature of *Schistosoma japonicum *infection is granulomatous inflammation induced by egg deposition, primarily involving the liver and intestines [2]. Schistosome eggs typically deposit in the liver parenchyma or intestines, while hilar bile duct granulomas represent rare ectopic lesions. Common neoplastic causes of hilar masses include hilar cholangiocarcinoma (the most frequent), metastatic lymphadenopathy, and hepatocellular carcinoma invading the hilum. However, the diagnostic challenge of hilar schistosomal granulomas lies in their clinical manifestations (such as painless jaundice, intrahepatic bile duct dilation, elevated cancer antigen CA19-9, and nonspecific symptoms), imaging features (such as hilar masses, bile duct wall changes, and PET-CT metabolic signals), and laboratory/epidemiological factors (such as serological false positives and insufficient clinician awareness of ectopic lesions in endemic areas), all of which highly overlap with hilar cholangiocarcinoma.

We report the case of an elderly male patient from an endemic area presenting with abdominal distension and jaundice, initially diagnosed with hilar cholangiocarcinoma but confirmed as schistosomal granuloma by postoperative pathology. The case enhances our understanding of the diagnosis and treatment of hilar schistosomal granulomas. This case is characterized by the rare location of the granuloma, with a series of symptoms and examination results leading to a preoperative misdiagnosis of hilar cholangiocarcinoma and subsequent radical resection, after which postoperative pathology revealed the true diagnosis of schistosomal granuloma.

## Case presentation

The patient was a 72-year-old male with a history of liver cirrhosis. He presented for medical treatment due to abdominal distension and yellow urine for 1 month. Physical examination on admission noted severe jaundice of the skin and sclera. The abdomen was flat and soft, with tenderness in the right upper quadrant and no rebound tenderness. There was no obvious tenderness or rebound tenderness in the rest of the abdomen. The liver and spleen were not palpable under the costal margin. Murphy's sign was negative, and there was no tenderness at the McBurney's point. There was no percussion pain in the liver area or both kidney areas. Shifting dullness was negative, bowel sounds were normal, and there was no edema in both lower extremities.

**Figure 1:**
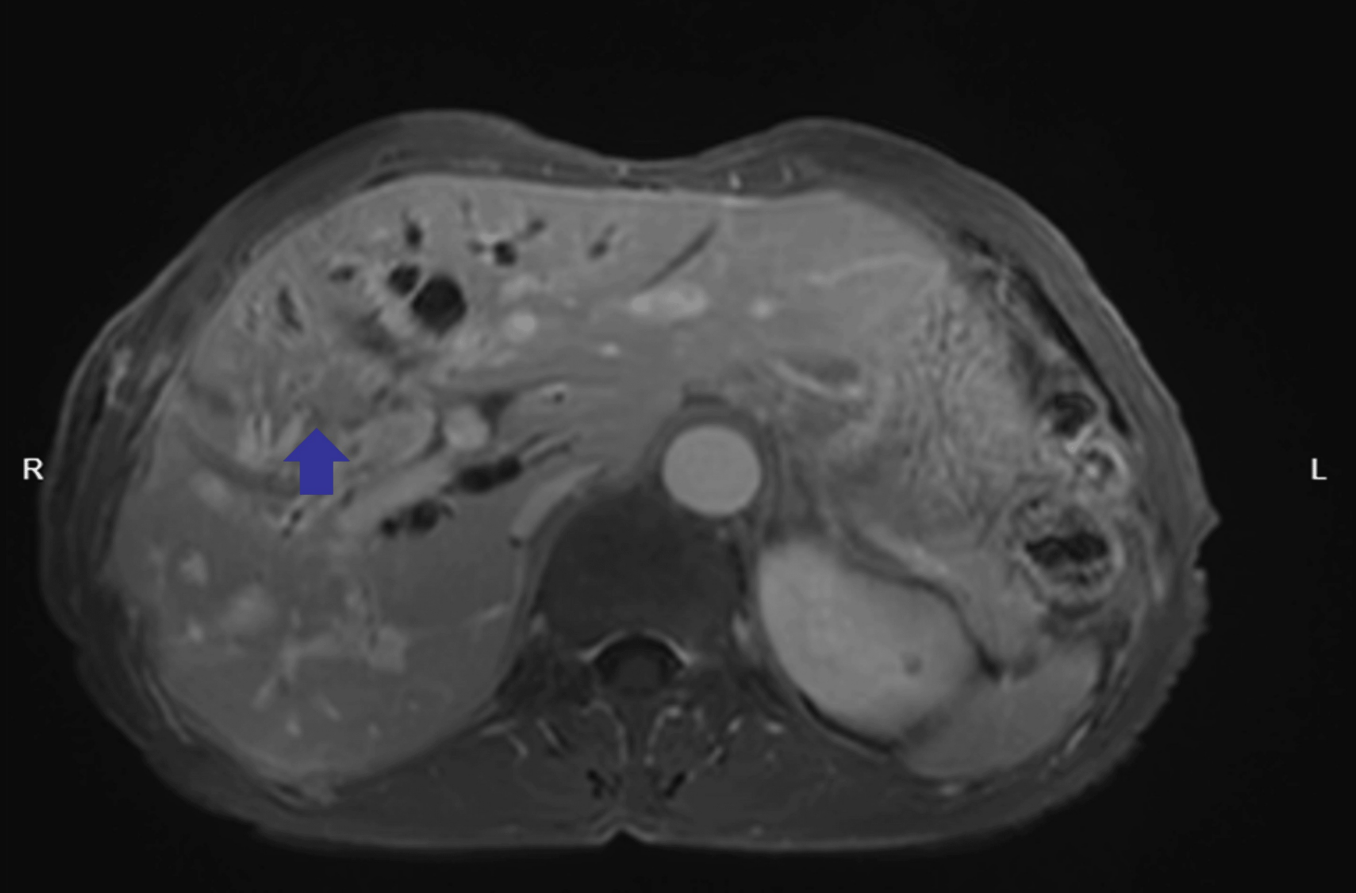
Contrast-enhanced MRI Contrast-enhanced MRI demonstrates a nodular lesion in the hepatic hilar region, showing slightly long T1 and T2 signals, slightly hyperintense signal on fat-suppressed imaging, measuring approximately 26 mm × 23 mm, with ill-defined borders (blue arrow).

**Figure 2:**
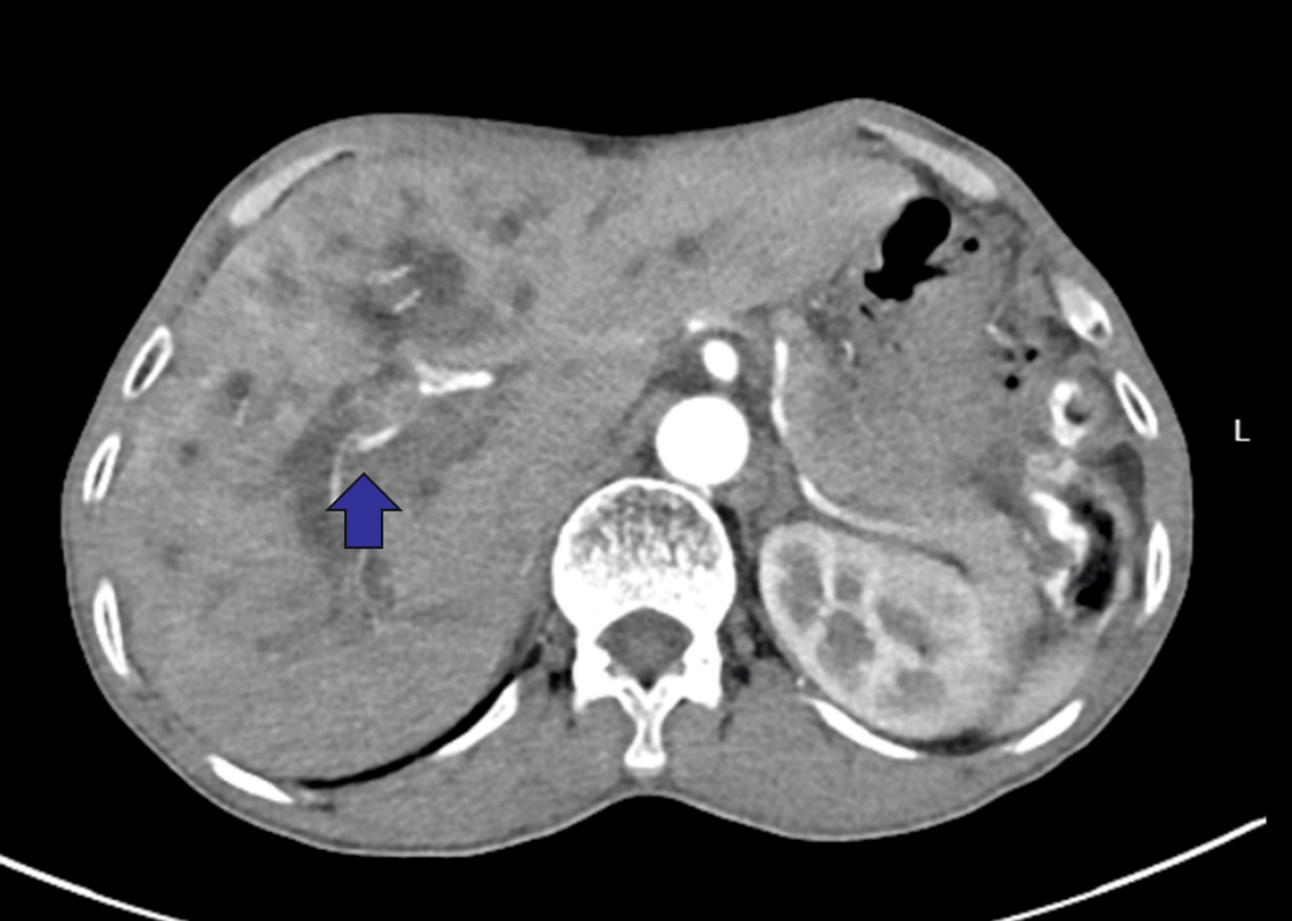
Contrast-enhanced CT showed dilation of intrahepatic and extrahepatic bile ducts (blue arrow)

Routine blood tests on admission (Table 1) showed a total bilirubin of 377.7 µmol/L and a direct bilirubin of 255.1 µmol/L, suggesting obstructive jaundice; CA19-9 was 103.20 U/mL, suggesting the possibility of malignancy. To clarify the cause of biliary obstruction, enhanced CT and MRI (Figures 1, 2) were performed on the second day: a 26 mm × 23 mm ill-defined mass was seen in the hilar region, with intrahepatic bile duct dilation and unclear display of the common bile duct. Initial consideration was hilar cholangiocarcinoma. Due to the severe obstructive jaundice of the patient, percutaneous transhepatic cholangioc drainage (PTCD) was performed under ultrasound guidance on the third day after admission. After the operation, symptomatic supportive treatments such as liver protection and fluid replacement were given.

**Table 1:** Laboratory blood tests on admission

Test Name	Result	Unit	Reference Range
Total Bilirubin	377.7	µmol/L	<21
Direct Bilirubin	255.1	µmol/L	<7
Indirect Bilirubin	122.6	µmol/L	<14
Total Bile Acids	325.2	µmol/L	<10
γ-Glutamyl Transferase (GGT)	345.3	U/L	10-60
Alkaline Phosphatase (ALP)	596.4	U/L	45-125
White Blood Cells (WBC)	2.80×10⁹	/L	3.5-9.5×10⁹
Neutrophil Percentage	33.10	%	40-75
Red Blood Cells (RBC)	2.85×10¹²	/L	4.3-5.8×10¹²
Hemoglobin (Hb)	97.0	g/L	130-175
Alanine Aminotransferase (ALT)	73.2	U/L	7-40
Aspartate Aminotransferase (AST)	71.3	U/L	13-35
Cancer Antigen (CA)19-9	103.20	U/mL	<37

To clarify the benign or malignant nature of the mass and the presence of metastasis, after communication with the family, PET-CT was completed on the fifth day. It suggested that the bile duct density at the confluence of the left and right intrahepatic bile ducts was slightly higher, and the glucose metabolism was persistently mildly increased (maximum standardized uptake value (SUVmax) 6.2). Since fluorodeoxyglucose (FDG) uptake with SUV < 2.5 is common in inflammation or low-grade tumors, the possibility of a biliary-origin malignant tumor was still considered.

**Figure 3:**
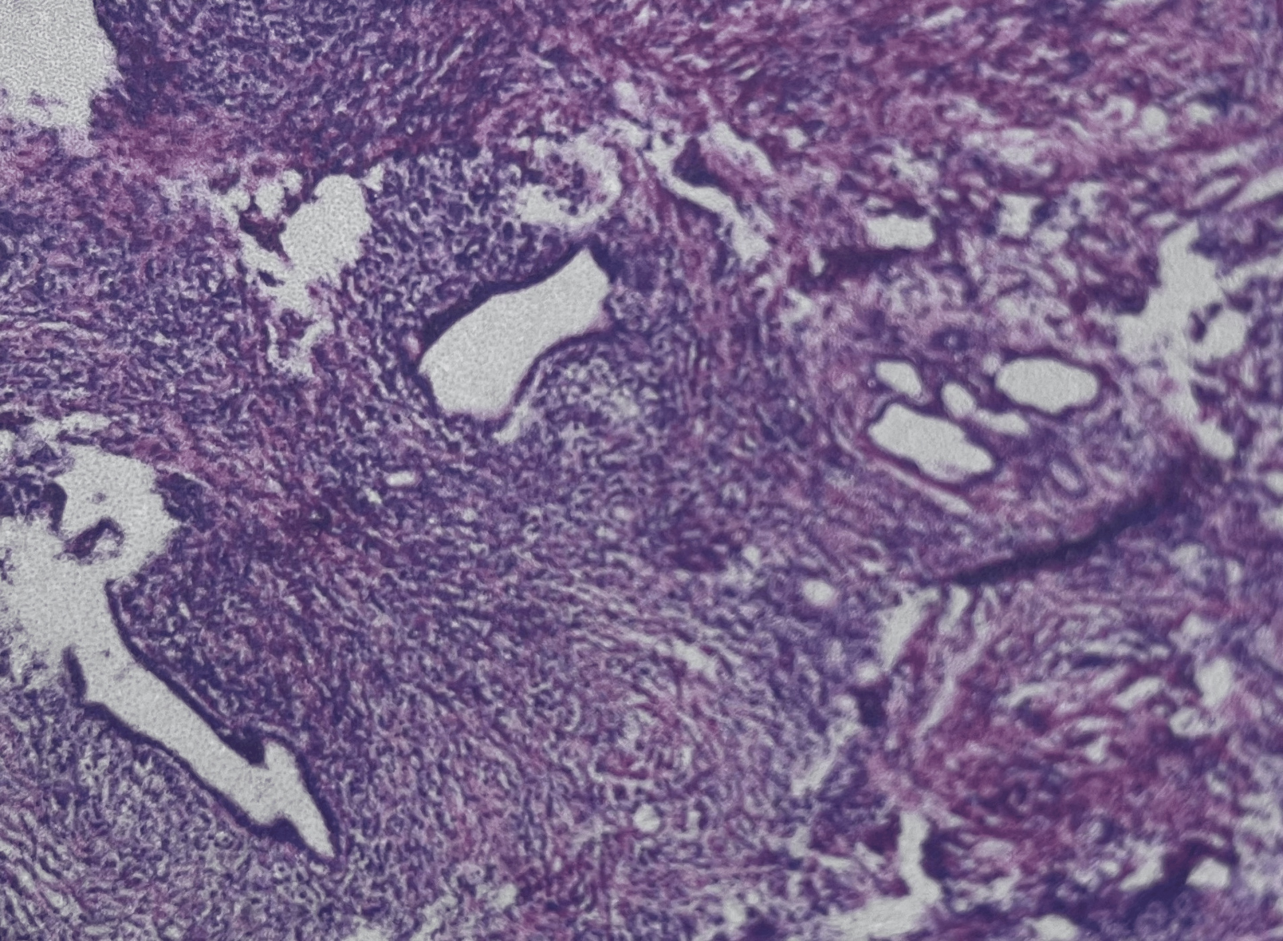
Pathological features of liver tissue Dilation of hilar bile ducts, proliferation of small bile ducts, interstitial fibrosis with massive inflammatory cell infiltration, and local calcified Schistosoma japonicum eggs were observed.

After completing the preoperative preparation, on the 15th day after admission, under general anesthesia, hilar bile duct tumor resection, partial liver resection of segments VB and VI, regional lymph node dissection of groups 8, 12, and 13, and choledochojejunostomy were performed. Postoperative pathological examination of the hilar liver tissue showed bile duct dilation, small bile duct hyperplasia, interstitial fibrosis with massive inflammatory cell infiltration, and local calcified Schistosoma eggs; meanwhile, reactive hyperplasia changes were seen in the lymph nodes of groups 8 and 13 (Figure 3). The PTCD tube was removed 2 months after surgery, and the liver function gradually recovered (total bilirubin decreased to 17 µmol/L, CA19-9 normalized). After 24 months of follow-up, there was no recurrence, and alpha-fetoprotein (AFP) remained negative.

## Discussion

The damage caused *by Schistosoma japonicum *to the human body involves four distinct developmental stages [3]. The pathological injury of *S. japonicum *infection is centered on egg deposition. During its developmental stages, eggs are deposited in the liver via the portal venous blood flow, inducing the aggregation of macrophages, eosinophils, and T lymphocytes to form granulomas that gradually fibrose [4-5]. Ectopic egg deposition in the skin, brain, and genitourinary system can lead to other clinical syndromes [6-8], but schistosome egg-induced hilar bile duct granulomas are extremely rare.

Clinically, this case presented with progressive jaundice and elevated CA19-9 levels. Contrast-enhanced MRI revealed a mass in the hilar bile duct. Although the patient had a history of schistosomiasis infection, risk factors for hilar cholangiocarcinoma include biliary parasitic diseases and recurrent biliary tract infections [9]. Early symptoms of hilar cholangiocarcinoma also include jaundice and abdominal discomfort [10], which further complicated our initial diagnosis. However, preoperatively, despite the patient's origin from an endemic area and history of schistosomiasis, clinical suspicion prioritized hilar cholangiocarcinoma, reflecting insufficient awareness of rare ectopic granulomas.

Serological tests (such as schistosome antibodies and the circumoval precipitin test) could have provided critical clues, but were not performed in this case, leading to a missed diagnosis. Additionally, endoscopic retrograde cholangiopancreatography (ERCP) with biopsy could directly obtain bile duct wall tissue, especially suitable for hilar lesions. ERCP can also perform bile drainage to relieve jaundice, serving both diagnostic and therapeutic purposes, but this approach was not adopted preoperatively.

Praziquantel exhibits highly selective toxicity against adult schistosomes by disrupting calcium ion channels in the parasite's cell membrane, leading to paralysis and subsequent clearance by the host immune system [11]. The efficacy of praziquantel on schistosomal granulomas remains unclear, though existing studies suggest it may have therapeutic potential [12]. Treatment of schistosomiasis primarily focuses on pharmacotherapy, with praziquantel effective against all stages and types of schistosomes. It also inhibits inflammatory cells and fibroblast proliferation, reducing granuloma formation. Therefore, the timely administration of praziquantel is recommended upon diagnosis of schistosomiasis.

In this case, the patient was not diagnosed with hilar bile duct schistosomal granuloma preoperatively and thus did not receive anti-schistosomal treatment. No reports have evaluated whether praziquantel combined with percutaneous transhepatic biliary drainage achieves satisfactory outcomes for obstructive jaundice caused by schistosomal bile duct granulomas. Further research is therefore warranted to determine the optimal treatment strategy for schistosome-induced obstructive jaundice. Although surgical resection was unnecessary, if radical surgery has been performed, completion of an anti-parasitic course postoperatively is required to reduce the risk of egg redeposition. For such patients, long-term follow-up should include liver function tests, parasitic serology, and imaging to monitor for new granulomas or biliary strictures.

## Conclusions

The patient in this case was from an endemic area and had a history of schistosomal cirrhosis but was misdiagnosed because the parasitic etiology was not considered preoperatively. Epidemiological history should be prioritized, especially for patients without clear high-risk factors for malignancy, such as alcoholism or primary sclerosing cholangitis. For cases suspected of hilar cholangiocarcinoma, it is recommended to detect schistosome eggs in bile via ERCP and perform biopsy or brush cytology of the bile duct wall lesions preoperatively. When the diagnosis is highly uncertain and there are no urgent indications for surgery, a short-term trial of praziquantel may be attempted to observe changes in jaundice and imaging. Although it cannot reverse the established granulomas, killing adult worms can prevent continuous egg deposition, which may help differentiate from malignant tumors based on therapeutic responses. Schistosomiasis typically involves the liver and intestines, but its ectopic manifestations are easily overlooked. In the future, systematic improvements in the ability to distinguish between benign and malignant lesions should be achieved through clinical education, the development of specific markers, and guideline formulation.
